# Preparation of asymmetric Janus hollow silica microparticle and its application on oily wastewaters

**DOI:** 10.1038/s41598-023-30269-9

**Published:** 2023-03-13

**Authors:** Hailong Zhang, Ting Qu, Hairong Wang, Weixing Wu, Fangfang Lu, Jiguang Ou, Genmin Zhu, Liangjun Gao, Longsheng Cheng

**Affiliations:** 1 Zhejiang Key Laboratory of Petrochemical Environmental Pollution Control, National-Local Joint Engineering Laboratory of Harbor Oil and Gas Storage and Transportation Technology, School of Petrochemical Engineering and Environment, Zhejiang Ocean University, Zhoushan, 316022 Zhejiang China; 2 National Engineering Research Center for Marine Aquaculture, Institute of Innovation and Application, Zhejiang Ocean University, Zhoushan, 316022 Zhejiang China; 3 Zhoushan Institute of Calibration and Testing for Quality and Technology Supervision, Zhoushan, 316000 Zhejiang China; 4 ENN (Zhou Shan) Natural Gas Pipelines Co., Ltd, Zhoushan, 316021 Zhejiang China

**Keywords:** Environmental sciences, Materials science

## Abstract

Janus nanoparticles have aroused the interest of scholars because of their highly efficient emulsification of spilled oils in wastewater. In this work, interfacially active Janus hollow glass microparticles (J-HGMPs) of asymmetric wettability were designed and synthesized in order to achieve more efficient separation of emulsified oil droplets from oily wastewater. Surface characteristic techniques such as FTIR, SEM, zeta potential and contact angle measurements had been employed to assess the amphiphilic surface properties of J-HGMPs. The oil removal/recovery performance of J-HGMPs in different oil–water systems and their interfacial activities were studied. As a particulate emulsifier, J-HGMPs could remove/recover > 96% oil from oil–water mixed phase. The results showed that J-HGMPs had strong interfacial activities and anchored firmly at oil/water interfaces. This high adsorption energy was also evaluated and verified via the calculation of Gibbs free energy. Overall, this study provided a novel and low-cost oil recovery method via a convenient buoyancy force that could be effectively applied in the treatment of oil spills while achieving the goal of benign and green environmental protection.

## Introduction

With the continuous development of offshore oil exploration and technology, marine pollution is becoming more and more serious. Offshore oil spills often caused by tankers and ship accidents have emerged as a major threat to the marine environment^1^. The spilled oils are dispersed at the sea surface to form a large oil film, hindering the dissolution of oxygen in the air and making it difficult for marine organisms to survive due to hypoxia^2^. At the same time, toxic substances in the oil slick seriously endanger the growth and survival of marine organisms, and also human health is indirectly threatened by enrichment through food chain^3^. Therefore, it is of vital important to develop advanced materials with environmentally benign, low-cost and recyclability, which can efficiently separate oil/water mixtures.

Up to now, many techniques have been applied to separate oil from water and these separation methods can be roughly divided into physical, chemical, physicochemical and biological methods^4–8^. Although some methods have already been applied in industry, there are still many drawbacks. For instance, dispersants, most of which are mixtures of chemical surfactants and organic solvents, are usually employed as an effective way to treat or eliminate oil spill pollution^9^. These dispersants are sprayed on the surface of oil films to reduce oil–water interfacial tension to form uniformly dispersed small oil droplets under the influence of wind and waves^10^, and then further to adsorb and disperse the oil/water mixture. Moreover, super wettable membrane and/or sponge materials have been developed and used as new materials to selectively remove oil slick and even surfactant stable oil droplets with high separation efficiency, the tiny oil droplets isolated from seawater is still not effectively treated and is often neglected, leaving the oil pollution problem unsolved^11,12^.

In recent years, amphiphilic Janus solid particles have attracted extensive attention from researchers because of their not only asymmetric chemical structure, versatile functions, and special surface physicochemical properties but also high public interests and high production efficiency, which have great potential in drug release, biology, emulsion stability, optical and electronic devices and other research and application fields^13–15^. Janus nanoparticles (NPs) with amphoteric surface structure have surfactant like properties. Due to their stability and strong interfacial interaction, they are the entities with opposite wetting properties, anchoring at the oil/water interface to form an oil-in-water emulsion^16–18^. In the application of enhance oil recovery (EOR), a novel nanofluid of amphiphilic Janus silica-based NPs has achieved the oil recovery by 15.7% for 100 mg/L oil solution^19^. Many studies have verified that amphiphilic NPs can easily anchor at the oil/water interface^20,21^, and their large surfaces make them adsorb more efficiently and stably at the oil/water interface as a stabilizer of emulsions^22–25^. The adsorption capability of interfacial active particles can be significantly enhanced if the NPs are with Janus properties, so that they have a strong stripping capacity for particulate emulsified oil droplets, so as to separate oil from oily wastewater effectively^26^. As the state of art, the interfacially active magnetic Janus NPs have been reported to adsorb the oil droplets^27^, and as well provide a good opportunity to recover adsorbed oil droplets via an external magnetic field, thereby achieving effectively to remove/recover waste oil from oily wastewater^28^. Over the past decade, great progress has been made in the research of NPs with various interfacial activities, easily recovery, and magnetic response. For example, magnetic NPs were modified by carboxymethyl cellulose (CMC) for hydrophilic portion and ethyl cellulose (EC) for lipophilic part^29,30^, and their efficiency in removing/recovering oil stains in oily wastewater was greatly improved. More recently, the approach of an emulsion interfacial polymerization was demonstrated to fabricate Janus micro-size entities with oleophilic styrene/divinylbenzene (St/DVB) and hydrophilic acrylic acid (AA) on concave and convex surfaces respectively^31,32^. Their micro-scaled lipophilicity surface was reported to be beneficial for recovering sub-micro size oil droplets (dissolved oil) in water. Their micro scale lipophilic surfaces may imply some advantages in removing tiny oil droplets from the aqueous phase. These magnetic Janus microparticles have significant capture ability, and can also quickly remove micro range oil droplets (emulsified oil) from the water phase, achieving a separation efficiency of up to 99%. Despite these advances, the cost of their synthesis as well as their practical applications could bring some difficulties to the magnetic recovery strategy for rough ocean surfaces, or under unexpected complex conditions. However, by smartly using amphiphilic hollow micron particles as particulate emulsifiers, the captured oil droplets can easily float on the ocean surface by virtue of their own buoyancy, which provides great convenience and practical operability for oil recovery and separation. To our best of knowledge, the application of this amphiphilic hollow micron particle so far has not been fully discussed.

Based on the concepts of self-recovery buoyancy and amphiphilic characteristics, a smart and concise strategy was designed to combine these two advantages into amphiphilic Janus-HGMPs via modifying the hemispherical surface of hollow silica glass microspheres with *N*-octadecyl trimethoxysilane to regulate their surface chemical components. Surface analysis methods such as FT-IR, Zeta potential, contact angle, and SEM were applied to analyze their surface properties. The adsorption principle of amphiphilic J-HGMPs at the oil/water interface and their emulsification of different oil strains in deionized water and artificial seawater (ASW) were also exposited systematically. This approach would be helpful to rationally utilize the amphiphilic hollow microparticle dispersant with its low-density advantage and promote innovation in the field of oil spill recovery.

## Materials and methods

### Materials and reagents

Hollow glass microparticles (HGMPs, diameter ~ 40 μm) were made of chemically stable borosilicate glass, with a density of 0.36–0.40 g/cm^3^ and an isostatic crushing strength of 38 MPa, provided by Sinosteel ANSHAN Mining Institute New Material Technology Co. Ltd. (China). *N*-octadecyl trimethoxysilane (ODMS, > 90%) and triethylamine (> 99.0%) were purchased from Macklin (China). Xylene (≥ 99.0%) and ethanol absolute (≥ 99.7%) were obtained from Shanghai Hushi Experimental Equipment Co., Ltd. (China). Acetone (≥ 99.5%) was provided by Ji'an Haomai Fine Chemical Industry Co. LTD (China). Diesel oil (> 98%) was procured from Aladdin Chemical Company, China. Crude oil (freezing point 23.0 °C, density 0.8552 g/cm^3^, viscosity 22.2 MPa/s at 50 r/min, 50 °C) was offered by a company from Shengli Oilfield, China. The seawater used was an artificial seawater (ASW) prepared freshly from the reference^23^. The pH value of ASW measured was 8.5. All chemical reagents were used directly without further purification. All water mentioned in our experiments was deionized water.

### Preparation of amphiphilic Janus-HGMPs

In this work, asymmetric Janus entities were prepared by using a one-step (the Pickering emulsion) method to decorate hydroxyl groups on their half surface with alkyl silanes^33^. A diagrammatic sketch of the preparation process of Janus HGMPs and fully lipophilic HGMPs was elucidated in Schematic S1. For the synthesis of amphiphilic Janus HGMPs, 1.25 g of HGMPs was fully mixed with 1.25 mL of water, and then added into a mixed solution of 40 mL, xylene, and 1 mL, ODMS. Mixed the solution evenly with magnetic stirring at 600 rpm for 3 min, and then added 1 mL, triethylamine into the mixture, continued stirring for further 40 min. The final reaction products were obtained by centrifugation, separation from solution, and rinsed twice with absolute ethanol, then finally dried in vacuum at 100 °C. After contacting with water partially, the portion surface of HGMPs remained hydrophilic (untreated hydroxyl groups), and the half surface unexposed to water reacted with ODMS to become lipophilic (alkyl) parts, therefore realizing amphiphilic Janus HGMPs. Completely lipophilic HGMPs were also prepared via the same steps as described above without the presence of water, this oleophilic product was marked as O-HGMPs, and cleaned HGMPs without treatment as W-HGMPs.

### Characterizations

The samples W-HGMP, J-HGMP and O-HGMP were analyzed by Fourier transform infrared spectroscopy (FTIR) (Nicolet FTIR 6700, Thermo Fisher, US) to detect their chemical composition and characterize typical chemical groups on the surface in the wave number range of 500–4000 cm^−1^. Zeta potential analyzer (ZetasizeNano ZS90, Malvern Instruments, UK) was used to measure the ζ potentials of various HGMP microparticles in water dispersion at a fixed scattering angle of 90 degrees. After uniform dispersion, optical images were observed and taken by a microscope (SetREO Discovery. V12, Carl Zeiss, Germany).

In order to determine their surface wettability of different HGMPs, their static contact angle (CA) measurements are carried out on a contact angle measuring instrument (OCA 15Pro, Data Physics ES, Germany). The surface morphology of samples was observed by scanning electron microscope (SEM). SEM (SUPRA-55, Zeiss, Germany) was employed to visualize the morphology of J-HGMPs, operating at 20 kV, equipped with an EDX microanalyzer. The dynamic interfacial tension of oil–water interface in the presence and absence of J-HGMPs was measured using pendant drop method on a contact angle measuring instrument (SZ-CAMB3). Diesel or crude oil is used as the drop phase, and deionized water is utilised as the environmental phase. During the measurement process, Janus-HGMPs (50 mg) particles were evenly dispersed in diesel or crude oil (10 mL) for 10 min, and 15 μL diesel or crude oil droplets were generated in the water phase. Record the dynamic interfacial tension within 600 s after the generation of oil droplets.

Residual oil content in water solution is measured and calibrated by infrared oil spectrometer (JLBG-126 Infrared Oil Spectrometer, Jiguang Technology Co., LTD, Jilin, China).

### Measurements of adsorption capacity, separation efficiency and reuse rate

4 mL of oil was added to ASW/DI water to prepare oil/water mixtures (40 mL). The mixtures were dispersed by 2 g Janus HGMPs, and then stirred with a magnetic stirrer at 600 rpm for 3 min. When their adsorption equilibrium is reached, the oil adsorbing particles are removed. Janus HGMPs has excellent separation performance for immiscible oil/water mixture, and no oil is observed in the resulting water. The oil content in various immiscible oil/water mixtures after separation was determined by infrared oil spectrometer^24^. Therefore, the calculation formula of separation efficiency (*E*) is:

1 $$E=\left(1000-CV\right)\times 100\%/1000,$$

where *C* is the oil concentration of mixtures after adsorption (mg/L), and *V* is the volume of mixtures (L). Reusability tests were also carried out, including each cycle adsorption and cleaning process. In the cleaning process, the settled and stable oily particles are thoroughly cleaned with toluene, the residual toluene is adsorbed with ethanol, cleaned repeatedly for three times, and then dried in a vacuum oven at 100 °C for 24 h. Each sample shall be cycled at least three times. The separation efficiency of Janus HGMPs for a series of immiscible oil/water mixtures can reach as high as 98.2%.

## Results and discussion

### Characterization of J-HGMP

**Figure 1:**
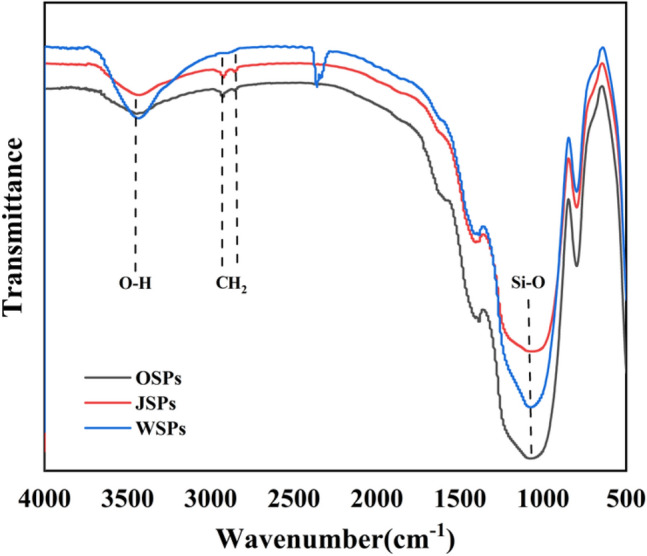
The chemical structure and composition of sample surfaces. FTIR spectra of (**a**) W-HGMPs, (**c**) amphiphilic Janus HGMPs, (**b**) O-HGMPs.

Amphiphilic Janus HGMPs were synthesized using a one-step and interfacial assembly method^33^. In order to corroborate the successful modification of SiO_2_ particles, the analysis of infrared spectroscopy was carried out. For FT-IR results, the FT-IR spectra of HGMPs, J-HGMPs and O-HGMPs were displayed shown in Fig. 1. The tensile vibration peak at 3440 cm^−1^ is the characteristic absorption peak of the O–H bond on the surface of silica micron particles, suggesting the predominant component (O–H bond) on the surface of HGMPs^34,35^, and the Si–O–Si antisymmetric stretching vibration corresponds to the absorption peak at 1039 and 1203 cm^−1^^36,37^. Compared with unmodified HGMPs, the C-H symmetric and anti-symmetric tensile vibration peaks at 2920 cm^−1^ and 2850 cm^−1^ of J-HGMPs indicate that silica micro-particles are modified by ODMS. The decay of the characteristic peak of the O–H bond on J-HGMPs and the appearance of the characteristic peak of the C–H bond indicate that J-HGMPs have amphiphilic properties. Hydrophobic hydrocarbon chain (C–H) was grafted on one side of the sphere, while hydrophilic hydroxyl group (O–H) remained on the other side, indicating that amphiphilic J-HGMPs had been successfully prepared. From Fig. 1, it is also found that that there is almost no characteristic peak of the O–H bond on the O-HGMPs, while compared with the J-HGMPs, the peak of the C–H bond is further enhanced, reflecting the unique hydrophobicity of the O-HGMPs^38,39^.

**Figure 2:**
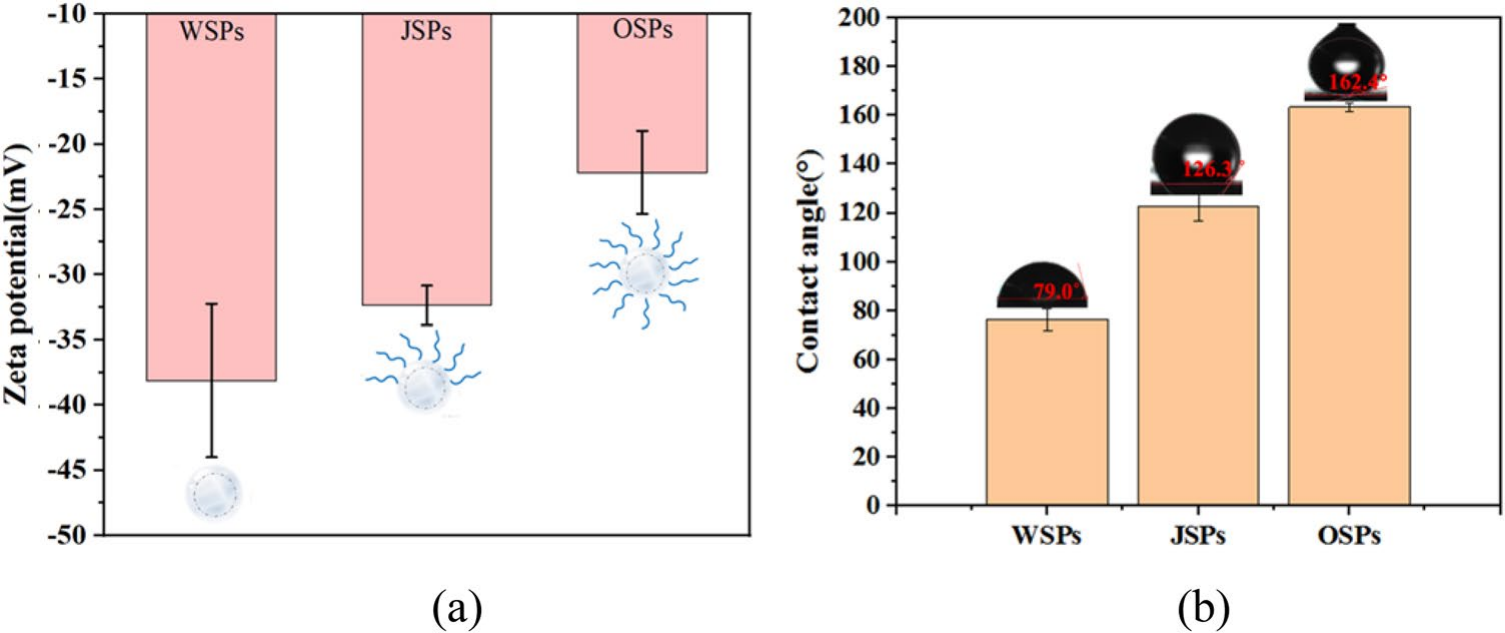
Surface property test. (**a**) ζ-Potential and (**b**) WCAs (water contact angles) of W-HGMP, J-HGMP and O-HGMP particles. The experimental data are repeated three times, and the error bar is a standard deviation.

To gauge the zeta potentials (ζ) of three types of HGMPs is another approach to characterize the change of surface properties of HGMPs after chemical group modification. As illustrated in Fig. 2a, the ζ potentials of three different HGMP particles has been measured. For the surface of unmodified particles, its ζ value measured at pH = 7 is − 35 mV, very close to the value of silica, which may be due to the large number of hydroxyl groups covering its entire surface^34,35^. However, for surfaces after being modified by ODMS, the negatively charged hydroxyl groups are replaced by two different covers of alkyl chains, partial and fully coverage, resulting in increases in the ζ potentials of J-HGMPs and O-HGMPs, which were measured to be − 31.5 and − 21.6 mV respectively. The values of ζ potential increase with the increase of alkyl chain modification levels. The results of different variations of their ζ potentials indicate that the two surface modification experiments of HGMPs are both successful. Due to the existence of excess hydroxyl groups on the particle surface, even for the surfaces of O-HGMPs fully coated with alkyl chain, their ζ potential is still negative.

Surface wettability is also an important characteristic of particle surface. The contact angle test directly reflects the hydrophilicity of the solid surface. Low contact angle indicates high wettability (hydrophilicity) and high surface energy. According to the angle value measured by the contact angle instrument, the smaller the contact angle is, the better the hydrophilicity is. The larger the contact angle, the better the hydrophobicity. Water contact angle (WCA) refers to the angle θ between the tangent line of gas/water interface and the solid-water boundary at the three-phase contact line of gas, liquid and solid. The WCA on the surface of solid material can quantify the wettability of a specific surface^40^. The measurement of WCA is used to characterize the diffusion degree of water on the surface of solid particles. Here, the WCA can be used to determine the ability of particles to contact with organic matter, and the stability of Pickering emulsion formation^41,42^. WCAs of different HGMP particles are measured to assess their wettability as exhibited in Fig. 2b^43,44^. The low WCA (76.5 ± 2°) measured for HGMP particles of unmodified sample shows its hydrophilicity because there present a large number of –OH groups on its surface, as indicated from the above discussion of FTIR spectrum results. After modification with a certain amount of ODMS, a large number of alkyl long chains have been grafted to the surface of J-HGMPs for partial coverage, which may reduce their hydrophilicity, resulting in the increase of the WCA of Janus HGMPs to 122.7 ± 3°; In the similar step during the preparation, the WCA of oleophilic O-HGMPs with fully coverage of alkyl chains can reach 163.4 ± 1.5°.

**Figure 3:**
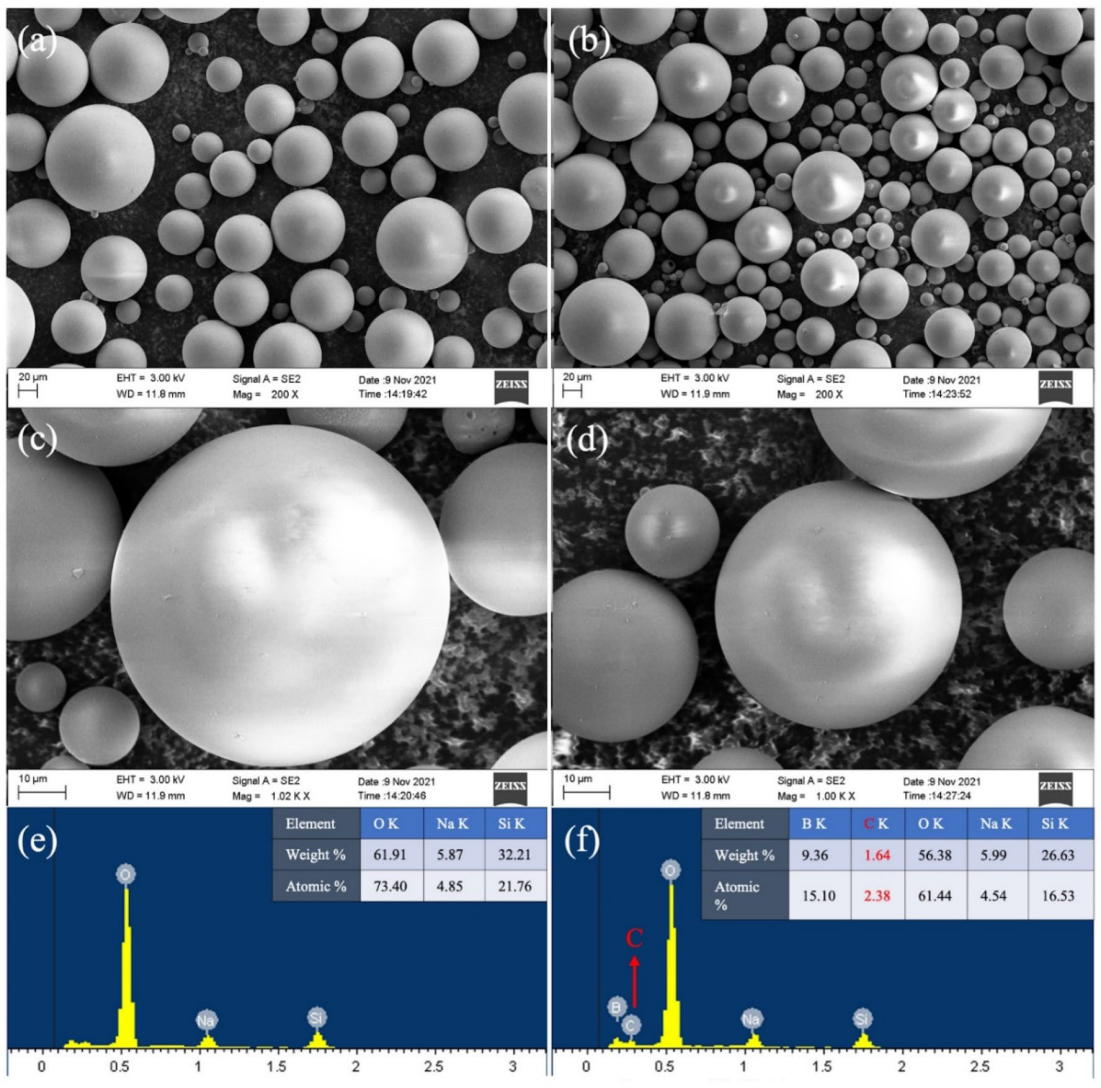
Surface characterization of samples. SEM images of W-HGMPs (**a**,**b**) and J-HGMPs (**c**,**d**) at different magnification, EDX of SEM diagrams of W-HGMPs (**e**) and J-HGMPs (**f**).

SEM micrographs of W-HGMPs and J-HGMPs samples are also measured and shown in Fig. 3. As can be seen from Fig. 3a–d, W-HGMPs and J-HGMPs are both smooth beads with good monodispersity, and the average particle size is about 40 ± 8 μm (diameter). The element composition and content analysis results of two samples, W-HGMPs and J-HGMPs, in Fig. 3e,f show that the surface of W-HGMP sample does not contain carbon, while the J-HGMP sample has a trace amount of carbon on its surface, which further proves that the surface of J-HGMP particles is modified with alkyl chain. The hydrophobic agent ODMS only reacts with the hydroxyl groups exposed on the half outer surface of HGMPs to decorate the surface a hydrophobic alkylsiyl monolayer on the asymmetric structure J-HGMPs^33^.

### Dispersion behaviors of different particles in water

**Figure 4:**
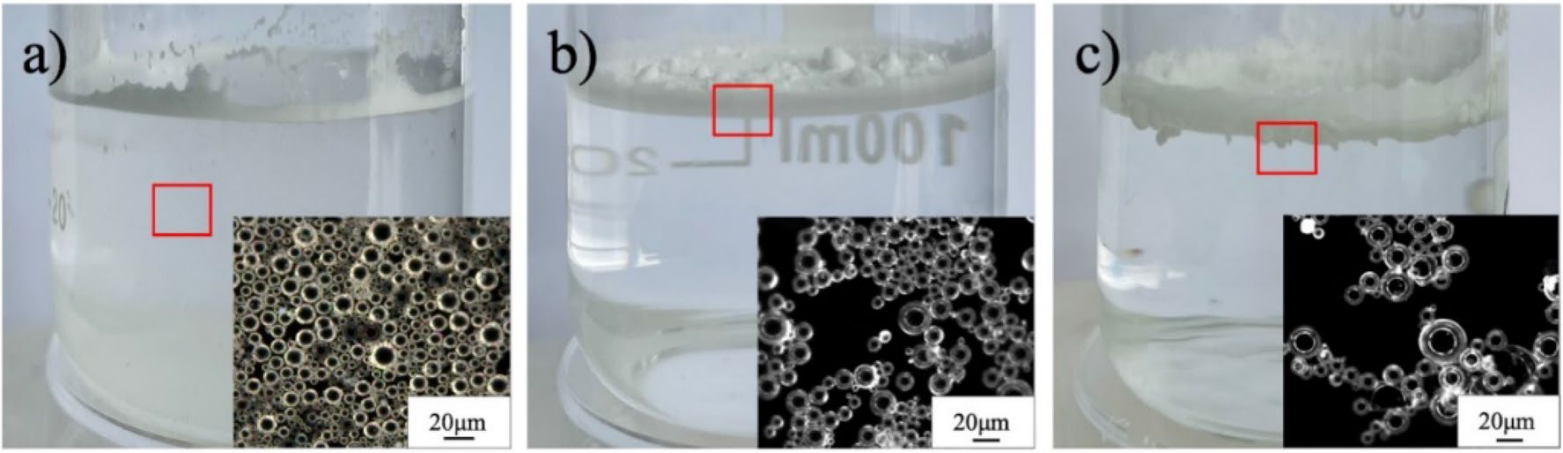
Dispersion performance trials. Dispersion behaviors of different particles, (**a**) W-HGMPs, (**c**) J-HGMPs, and (**b**) O-HGMPs in water phase.

Dispersion behaviors of as-prepared J-HGMPs compared with W-HGMPs, and O-HGMPs in water are illustrated in Fig. 4. As shown in Fig. 4a, hydrophilic W-HGMPs appear to stay on the top of water and some are adsorbed on the beaker wall. Unlike hydrophilic particles, hydrophobic O-HGMPs arrange themselves on the water surface with a very loose and irregular shape (Fig. 4c). Due to their hydrophobic surface and lighter specific gravity than water, they float and gather irregularly at the top of water surface. Therefore, those two particles both with a surface of uniform wettability cannot secure or maintain a stable emulsion. Compared with above two particles, amphiphilic J-HGMPs illustrate a stable and uniformly arranged dispersion layer, as shown in Fig. 4b, forming a better dispersion on the water surface. Observing under the microscope, they are dispersed evenly on the water surface as shown in the inserted image in Fig. 4b. These results have indicated that J-HGMPs have advantages over other two particles, W-HGMPs and O-HGMPs, in achieving the stable Pickering emulsion.

**Figure 5:**
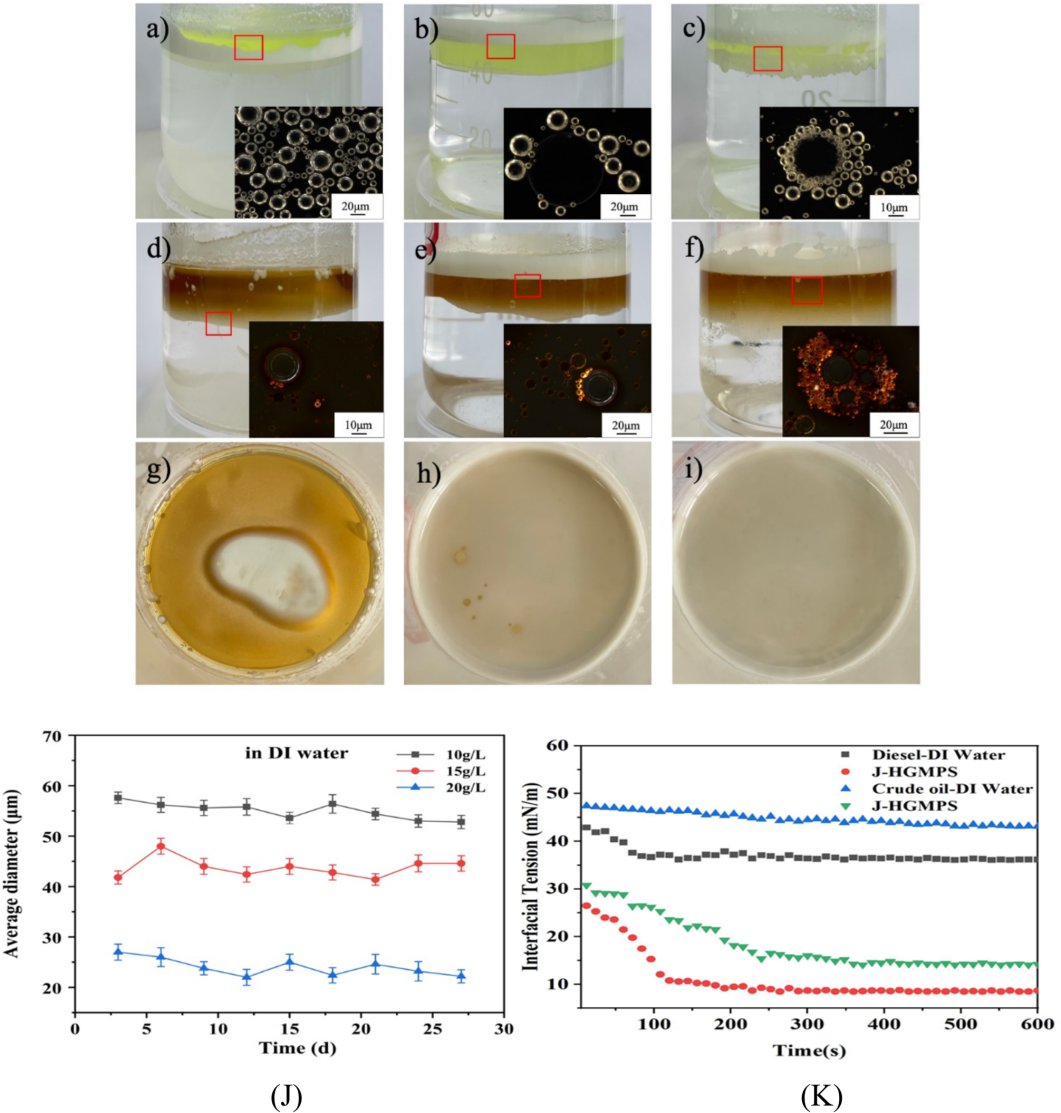
Dispersion experiments. Dispersion behaviors of various HGMP particles in oil–water mixtures: for diesel-water, (**a**) W-HGMPs, (**b**) O-HGMPs, (**c**) and J-HGMPs; for crude oil–water, (**d**) W-HGMPs, (**e**) O-HGMPs, (**f**) and J-HGMPs; and top view, (**g**) the top view of (**d**), (**h**) the top view of (**e**), (**i**) and the top view of (**f**) J-HGMPs; (**j**) average diameter of oil droplets of Janus HGMPs particles with different concentrations at the interface between diluted crude oil and deionized water (volume ratio of diluted crude oil to deionized water: 1:9); and (**k**) Interfacial tension of Janus HGMPs particles at different oil–water interfaces (diesel oil deionized water interface and diluted crude oil deionized water interface). The experiment was repeated three times, and the relative experimental error was ± 1%.

In order to further understanding their amphiphilic characteristics, dispersion performance of J-HGMP particles in diesel/crude oil water mixtures in comparison with W-HGMPs and O-HGMPs particles has been carried out. As shown in Fig. 5a, when W-HGMPs particles are added to the emulsion of diesel and water, a large number of W-HGMPs particles appear randomly to coalesce on the water surface because of their lighter specific gravity. As shown in Fig. 5b, due to hydrophobic interaction, lipophilic O-HGMP particles are attracted into the oil phase, then they converge and accumulate on the top of oil surface, forming irregular dispersion. In contrast, amphiphilic J-HGMPs particles clearly accumulate at the interface of oil/water mixtures, as exhibited in Fig. 5c. This phenomenon of amalgamation on the oil/water interface explains their amphiphilic characteristics. These results are consistent with the observation in literature that the dispersion behavior of amphiphilic Janus HGMP particles in various systems appears more stable^43^.

As shown in Fig. 5d, side view and Fig. 5g, top view, for crude oil water mixtures, W-HGMP particles appear to not only disperse into the aqueous phase, but also converge to the top surface due to their buoyancy. On the other hand, lipophilic O-HGMP particles disperse into the crude oil phase, and float on the surface of crude oil as well, due to their lighter specific gravity, as shown in Fig. 5e, side view. From the top view (Fig. 5h), it can be seen that some sporadic oil droplets appear on the surface. In the same system, amphiphilic J-HGMP particles also disperse, but adsorb at the oil/water interface, Fig. 5f (side view). During mixing process, crude oil droplets may form and scatter in the aqueous phase. However, after mixing, no oil droplets can be observed on the surface as illustrated in Fig. 5i (top view). It may indicate that the oil droplets are completely stripped by amphiphilic particles (J-HGMPs). From these results of stripping actions, it can be inferred that amphoteric J-HGMPs have strong adsorption capacity, orientation ability and surface activity at the oil/water interface, which can effectively abate the interfacial tension at the oil/water interface, thus achieving better stable emulsion.

In order to study the influence of different concentrations of Janus particle on anchoring behaviors toward oil droplets, average diameters of oil droplets of Janus HGMPs particles with different concentrations at the interface between diluted crude oil and deionized water have been measured. As shown in Fig. 5j, small changes in the average diameters of oil droplets can be visualized. It can be confirmed that the emulsification effect of Janus HGMPs particles on oil increases with the increase of Janus HGMPs particle concentration. By adding Janus HGMPs particles with different concentrations, it has been found that the oil droplets on the surface of deionized water are rapidly agglomerated with Janus HGMPs particles, and the oil droplets on the surface are significantly reduced.

The interfacial tension between oil and water phases of Janus HGMPs particles in different oil phases was measured. The experiment results are shown in Fig. 5k. It can be seen from Fig. 5k that the oil–water interfacial tension of Janus HGMPs particles reaches the lowest value for the diesel phase, only 7.83 mN/m, which is less than the interfacial tension of Janus HGMPs particles for the diluted crude oil deionized water interface, 13.6 mN/m. The reduction of oil–water interfacial tension makes tiny oil droplets easy to be stripped from oily wastewater^32^.

### Janus HGMPs application for removal/recovery of oily wastewaters

Janus particles have the advantage of superior interfacial activity and therefore is expected to strip oil from oily wastewater more effectively^24^. A large number of hydrophobic alky groups grafted on their micro-sized surface may offer higher potential to capture tiny oil-droplets out of water phase^31^. In order to verify this hypothesis, microscale J-HGMPs are applied to the capture of oil from oily wastewater. Firstly, the interfacial activity of J-HGMPs in complex oily wastewater systems (such as synthetic surfactants stabilized crude oil emulsion and diesel oil emulsion) has been studied.

**Figure 6:**
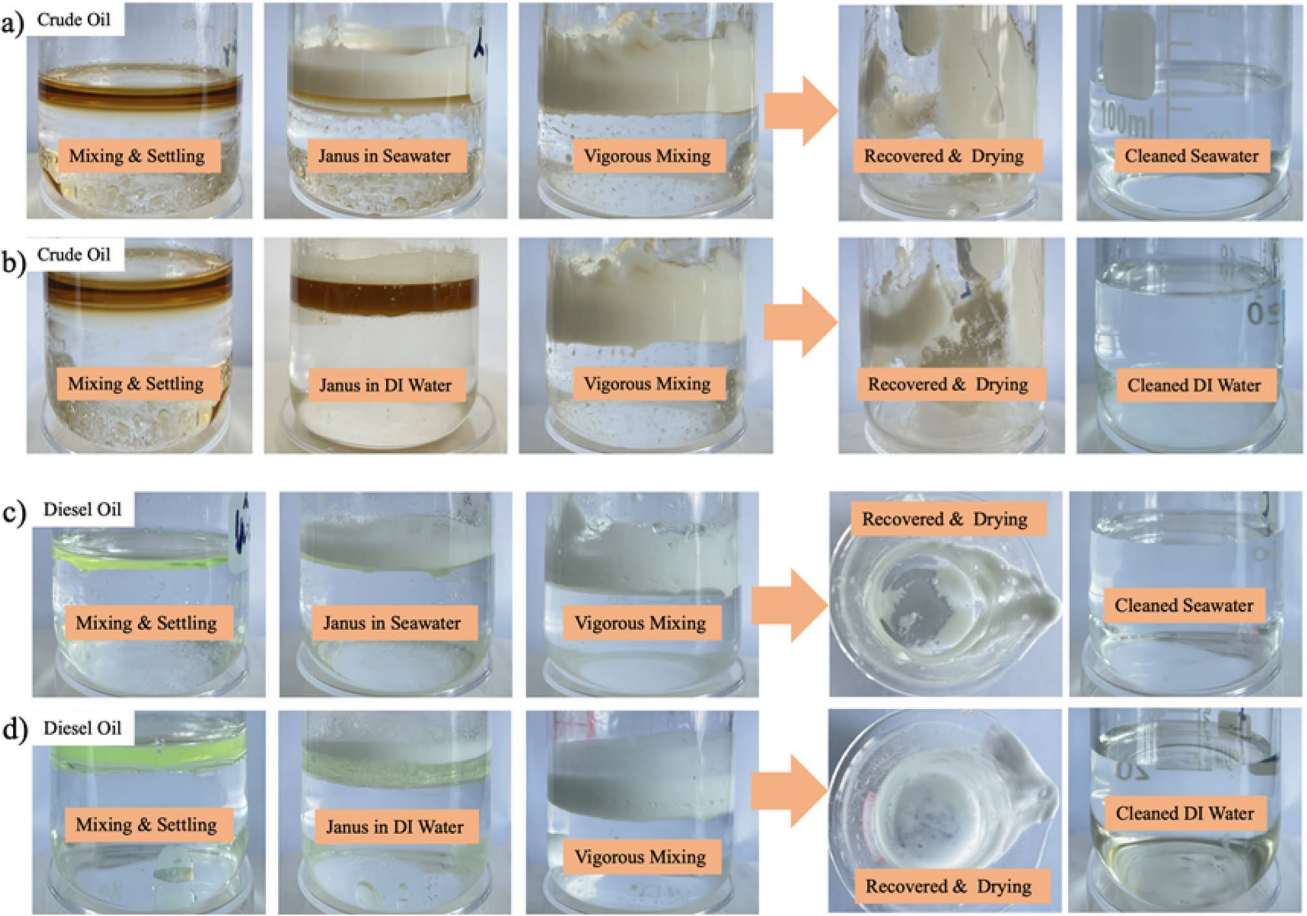
Application of EOR. Janus HGMPs application for removal/recovery of oily wastewaters: (**a**) diluted crude oil in ASW, (**b**) diluted crude oil in deionized water, (**c**) diesel in ASW; and (**d**) diesel in deionized water. The concentration of J-HGMPs in the corresponding aqueous phase is 30 mg/mL.

Figure 6 illustrates the dispersion behavior of J-HGMPs in removing/recovering crude oil or diesel oil from as-prepared oily wastewater: mixtures of crude oil and ASW (Fig. 6a), mixtures of crude oil and water (Fig. 6b), diesel oil and ASW (Fig. 6c) and diesel oil and water (Fig. 6d). Oily wastewater is formed by fully mixing oil (crude oil or diesel oil) and liquid (ASW or water) in a volume ratio of 1:9 (V_oil_/V_water_) via stirring. After 2 h settling for the oily wastewater, no obvious oil–water phase separation appears in the oily wastewater, suggesting that as-prepared oil–water emulsion has a relatively high stability. However, after adding J-HGMPs to the emulsion and sufficient mixing, the oil phase was observed to be fully stripped by J-HGMPs, and then oil droplets embedded with J-HGMPs were separated from water phase with a 200-mesh sieve, as shown in Fig. 6a,b, water phase becomes clear. Similarly, for diesel oils either in ASW or in water, diesel oil particles or oil phase were thoroughly wrapped by J-HGMPs (as illustrated in Fig. 6c,d while the milky white particles float on the liquid surface, and the yellow green colour of diesel oil is completely disappeared. The stability of emulsified oil droplets is often subject to the chemical properties of mixtures of water and oil, and their surrounding environments^31^. As indicated in Fig. 6b, with relatively high concentration of salts in seawater (compared to water), the interfacial activity of their components (for instance, asphaltene and/or natural surfactant in crude oils) can be greatly boosted. Therefore, in artificial seawater or seawater, it is very difficult for ordinary surfactants to deal with oil spills^32^. However, our experimental observation illustrates that J-HGMPs are ready to form stable emulsion.

As illustrated in Schematic S2, J-HGMPs suspension was prepared by dispersing J-HGMPs in water or ASW, and then added to oily wastewater (a). With superior interfacial activity, J-HGMPs can firmly anchor at the oil/water interface, and disperse the floating oil on the water surface. In addition, due to their lighter specific gravity (0.40 g/cm^3^), J-HGMPs firmly anchor oil droplets to form a larger oil droplet embedded in the package of J-HGMPs, and keep them floating on the water surface to facilitate recovery and removal from aqueous phase (b). Therefore, the oil droplets can be separated/recovered from the oily wastewater to obtain a clean water phase, which contributes a safe, low-cost, and benign strategy for the treatment of oily wastewater (c–e). Then, the separated J-HGMPs-oil particles were then retrieved and collected, and then cleaned thoroughly with toluene, ethanol was used to adsorb residual toluene to regenerate J-HGMPs (f) for subsequent application (g).

### Efficiency of Janus HGMPs in oil removal/recovery

**Figure 7:**
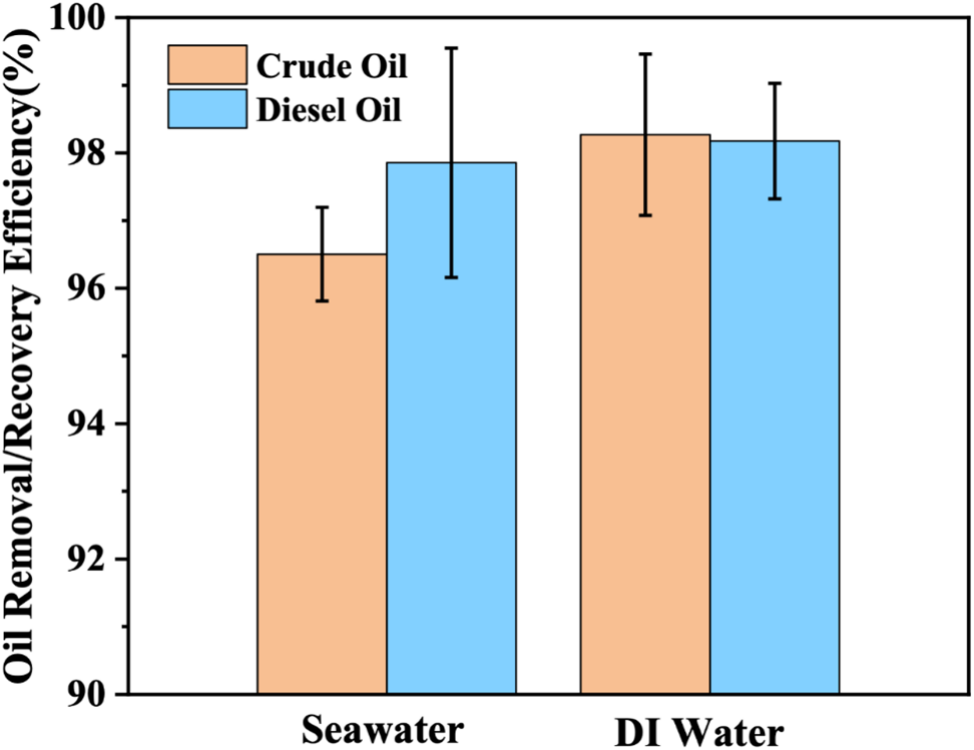
Oil removal/recovery efficiency test. Efficiency of using Janus HGMPs to remove/recover crude oil and diesel oil from oily wastewater. The oil removal/recovery experiment is repeated three times, and the error bar is a standard deviation.

The separation efficiency of oil–water mixtures in different systems was also investigated. The oil content of oily wastewater before and after oil removal/recovery was measured by infrared oil spectrometer. The efficiency of crude oil removal/recovery from ASW and water using J-HGMPs was measured and calculated to be 96.5 ± 0.69% and 98.27 ± 1.19% respectively; and the efficiency of diesel oil removal/recovery from ASW and water was 97.85 ± 1.69% and 98.18 ± 0.85% respectively, as illustrated in Fig. 7. These data showed that salinity and pH in ASW might interfere with the anchoring ability of J-HGMPs to oil phase and affect the oil recovery to varying degrees. Through the comparison of the two systems, the removal efficiency of J-HGMPs in ASW was slightly lower than that in water. However, under practical marine conditions, components in seawater were complex, including various inorganic salts, pH of seawater (8.5), surfactants, organic compounds, proteins and even bacteria, organic pollutants usually coexist with oil droplets^45^. Furthermore, the measurement of water content in the recovered oil illustrated that the water content in the recovered oil-rich phase was less than 0.5 wt%. Because the wastewater concentration is usually very low, the recovered oil is of satisfactory quality and can be used for subsequent refining. It is important to note that due to the wetting of the remaining oil film on the hydrophobic portion of Janus HGMPs, the oil removal/recovery efficiency from the oily wastewater is less than 100% and this portion of oil will be further recovered and recycled. The experiment results indicate that even if the volume fraction of oil is 0.25%, effective separation can be achieved in all cases. He et al. reported that the obtained oil removal/recovery rates in ASW and water were 95.1% and 96.7%, respectively^32^.

In addition, as indicated in Fig. 7, after three cycles of separation, its efficiency has hardly changed, suggesting that as-prepared J-HGMPs are stable, and J-HGMPs has good recyclability. This study clearly shows that after surface modification, J-HGMPs obtain strong interfacial activity, can firmly anchor at the oil/water interface, and render the oil removal/recovery rate greater than 96.5%. Because their specific gravity is lighter than water, oil droplets embedded with J-HGMPs become floating above the water surface, therefore, their collection and oil recovery become convenient as well. By this way, J-HGMPs can be reused efficiently in subsequent oil removal/recovery applications without complex regeneration. In sum, the utilization of Janus HGMPs can realize the rapid and effective separation of oil droplets from oil wastewater without the aid of any magnetic force, and may have strong and wide applicability in treating different types of oily wastewater in the field.

In order to better illustrate the relationship between the binding interaction performance of J-HGMPs with oil molecules and its interfacial activity, the mass of particles at the oil–water interface can be estimated, and this value can be calculated by finding the results of total mass of particles added minus the mass of particles in the aqueous phase. The mass of Janus HGMPs remaining in the aqueous phase after oil recovery was measured three times, and the obtained values were listed in Table 1. The results indicate that the mass of Janus HGMPs in the aqueous solution after oil recovery is less than that of M-Janus NPs in the literature^32^. This indicates that more Janus HGMPs (91.2% for crude oil and 92.5% for diesel oil in ASW) are attached to the oil/water interface than M-Janus NPs (88.9% for crude oil and 85.5% for diesel oil in ASW).

**Table 1:** Masses of J-HGMPs at the oil–water interface and collected in the aqueous phase after oil recovery are listed and compared with M-Janus NPs.

Oily wastewaters	Janus HGMPs^a^			M-Janus NPs^b^	
	At oil–water interface (mg)	In aqueous phase (mg)		At oil–water interface (mg)	In aqueous phase (mg)
Crude oil					
ASW	91.2 ± 1.5 (91.2%)	8.8 ± 1.5		133.2 ± 2.3 (88.9%)	16.8 ± 2.3
DI water	93.6 ± 2.1 (93.6%)	6.4 ± 2.1	Tap Water	137.3 ± 1.5 (91.5%)	12.7 ± 1.5
Diesel oil					
ASW	92.5 ± 1.2 (92.5%)	7.5 ± 1.2		128.3 ± 1.7 (85.5%)	21.7 ± 1.7
DI water	94.3 ± 2.3 (94.3%)	5.7 ± 2.3	Tap Water	130.7 ± 3.4 (87.1%)	19.3 ± 3.4

The measurements of the particle mass collected in the aqueous phase after oil recovery were repeated three times, and the error range was one standard deviation of the measurements. ^a^Data were collected in this work; ^b^Data were from Ref.^32^.

### Janus HGMPs in contribution to COD

**Figure 8:**
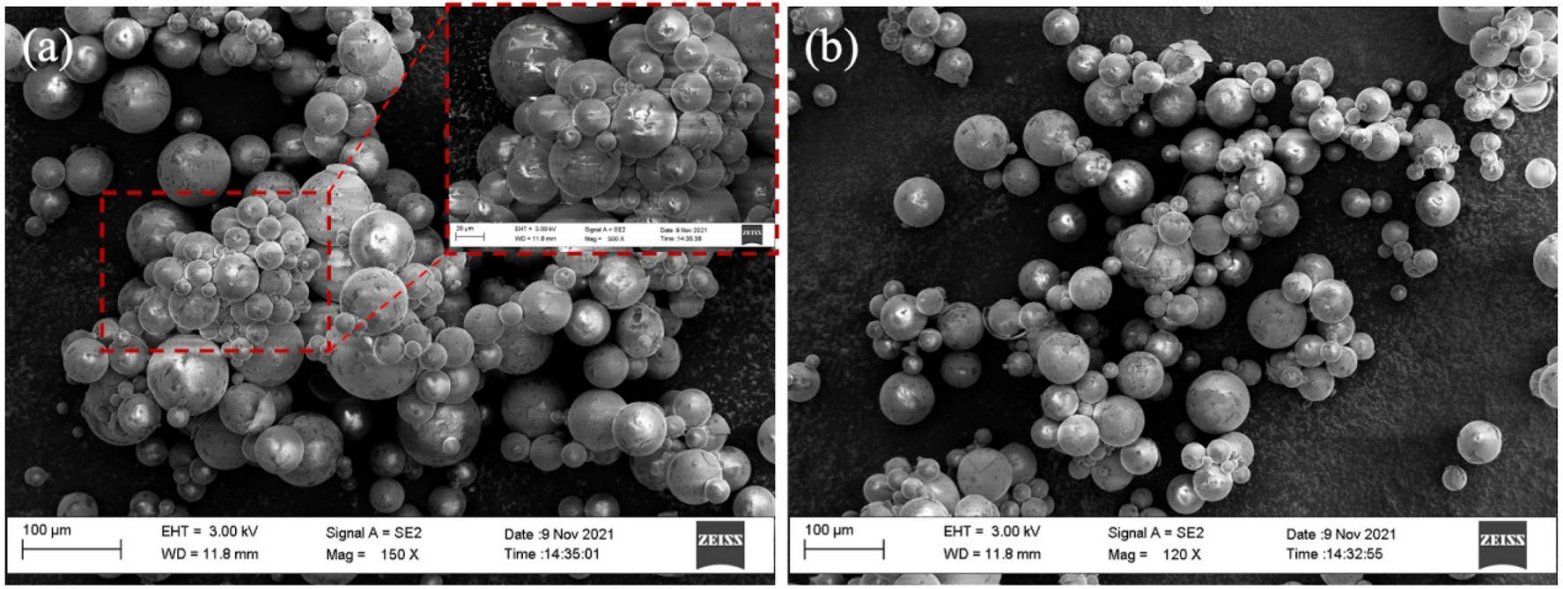
Morphologies of aggregated J-HGMPs after oily wastewater treatment. SEM images of aggregated J-HGMPs (**a**,**b**) after treating oily wastewater, image inserted for higher magnification.

Chemical oxygen demand (COD) is an important control index of petrochemical wastewater (PW) emission. As an extension application of J-HGMP samples, it is found that the COD values also decrease after removing oil phase from industrial oily wastewater. Table 2 shows that the COD value of the collected oily wastewater in comparison with those of the oily wastewater treated by J-HGMPs in different qualities. As shown in Table 2, the COD values are measured and listed. It is indicated that the as-prepared amphiphilic particles not only have the ability to remove oils from the oily wastewater, but also contribute to reducing the value of COD in the oily wastewater, which decreases from 236 to 144. These results may indicate some new potential applications. In particularly, for small organic molecules, such as methy tert butyl ether (MTBE), MTBE may form hydrogen bonds with water molecules and dissolve in water, making routine treatments/removal difficult in order to depress the COD value. After the removal treatments, J-HGMP samples with oil-droplets attached were collected, and their morphologies of aggregated J-HGMPs after treating oily wastewater were examined via SEM images, as exhibited in Fig. 8. After the crude oil suspension was treated with J-HGMPs, the appearances of J-HGMP clusters emerged immediately after their adsorbing oil droplets. As seen from high-magnification SEM image (Fig. 8a (inserted)), the accumulation shapes of J- HGMPs assemblies showed the formation of J-HGMPs aggregates with oil droplets embedded. Therefore, the morphologies from SEM images demonstrated that after being anchored by J-HGMPs, crude oil droplets and even small organic molecules could be encapsulated within J-HGMP clusters.

**Table 2:** Measurements of oily wastewater COD before/after treatment with Janus HGMPs.

Samples	pH	Total NH_3_ + N	COD	Remove rate of COD
Oily wastewater 100 mL	6.96	12.03	236	–
Oily wastewater 100 mL + J-HGMPs, 0.125 g	7.16	9.16	196	16.9%
Oily wastewater 200 mL + J-HGMPs, 0.250 g	6.66	10.77	144	39.1%

### Mechanism discussion

Unlike surfactants (usually small organic molecules), J-HGMPs show some significant advantages. First. because they are solid particles independent of the aqueous phase, and because of their large size, they have large interfacial adhesion energy^46^, which suggests that once small organic molecules are adsorbed on their interfaces, they will be trapped and separated from the aqueous phase, which also leads to their diffusion back to the liquid phase relatively slow, so this Pickering emulsion shows super stable characteristics. Second, because the specific gravity of J-HSMPs is relatively small (0.40 g/cm^3^), the more oil droplets captured by J-HSMPs and the larger their size, the grater buoyancy, and the more conducive they will be to recovery^47^.

The calculation of adsorption energy (ΔG) can be used to assess the surface activities of J-HGMPs, which is described as the free energy to gain for an entity from the oil/water interface to the bulk aqueous (water) phase, i.e., E_w_ − E_interf_. Although it is an approximate measurement of the actual effect, it is still an important index to gauge the surface activity and adsorption capacity of particulate emulsifiers^48^. Further calculations (Supplementary information) can help us understand that Janus particles at the micron scale may have better adsorption activity than that at the nano scale. For J-HGMPs, the *ΔG*_*2*_ value of particles desorbing from the oil/water interface into water phase can be obtained by Eq. (2):

2 $$\Delta {G}_{2}=\uppi {R}^{2}\left(2{\upgamma }_{w}Cos{\theta }_{2}+{\upgamma }_{o-w}\right).$$

For above equations, *R* is the radius of J-HGMPs, *θ*_*2*_ is the contact angle obtained via the water phase, and γ_*w*_, γ_*o-w*_, γ_*w-pho*_, γ_*o-pho*_, and γ_*w-phi*_ are the surface tension of water, interfacial tension between of oil and water, water and the lipophilic part of J-HGMPs, oil and the lipophilic part of J-HGMPs, water and the hydrophilic face of J-HGMPs, respectively.

Substituting the values into Eq. (2), it is found that the desorption energy of J-HGMPs (*ΔG*_*2*_) at the oil/water interface is strong, which is consistent with the observed the relatively stable emulsions, as illustrated in Fig. 5c,f. From Eqs. (2) and (S6), it can be suggested that the ΔG value of micron JSPs is at least three orders of magnitude higher than that of corresponding nano JSPs. Compared with nano-competitors, using Eq. (S6), we obtained 1.86 × 10^–14^ J, if J-HGMPs was in the system^48^, the results would be 1.05 × 10^–10^ J. It is worth mentioning that the ΔG value of micron JSPs can be 3–4 orders of magnitude higher that of their nano competitors. This result could imply more research activities on the application of Janus microparticles.

## Conclusions

In this paper, the distinct assembly characteristics and response of amphoteric Janus HGMPs at oil–water interfaces have been studied. The emulsion experimental results illustrate that Janus HGMPs show the effective dispersion of crude oil in ASW/water. Through its own buoyancy, more than 96.5 ± 0.69% oil can be removed/recovered from oily wastewater. Our recovery tests suggest that Janus HGMPs are easy to recover and reuse, and the regeneration process is simple without complex technique involved. The higher interfacial adsorption energy of Janus HGMPs indicates that they are capable of capturing small organic molecules in oily wastewater, thus contributing to the reduction of COD value. Furthermore, in comparison with magnetic Janus NPs, it is the more economical and effective way in recovering amphiphilic Janus HGMPs. Compared with conventional molecular surfactants, this microscale hollow particulate emulsifiers not only provide a more stable and reusable method, but also have the advantages of cost-effective and non-toxic to the environment. This research may open up a new and practical route for future oily wastewater treatment and the clean-up of industrial oily wastewater.

## Supplementary Information

Supplementary Information.

## Data Availability

All data generated or analyzed during this study are included in the manuscript and supplementary information.
