# Supplementary material for: Preparation of asymmetric Janus hollow silica microparticle and its application on oily wastewaters
Source: Sci Rep. 2023 Mar 13;13:4135. doi: 10.1038/s41598-023-30269-9 (PMC10011370; doi:10.1038/s41598-023-30269-9)
Supplement: Supplementary file 1 — Supplementary Information. [file 41598_2023_30269_MOESM1_ESM.docx]

**Supporting Information**

**Preparation of asymmetric Janus hollow silica microparticle and its application on oily wastewaters**

Hailong Zhang ^1^, Ting Qu ^2^, Hairong Wang^3^, Weixing Wu^1^, Fangfang Lu^3^, Jiguang Ou^4^, Genmin Zhu^1^, Liangjun Gao^1 *^, Longsheng Cheng ^4^

^1^ Zhejiang Key Laboratory of Petrochemical Environmental Pollution Control, National-Local Joint Engineering Laboratory of Harbor Oil and Gas Storage and Transportation Technology, School of Petrochemical Engineering and Environment, Zhejiang Ocean University, Zhoushan, Zhejiang, 316022, China;

^2^ National Engineering Research Center for Marine Aquaculture, Institute of Innovation & Application, Zhejiang Ocean University, Zhoushan, Zhejiang, 316022, China;

^3^ Zhoushan Institute of Calibration and Testing for Quality and Technology Supervision, Zhoushan, Zhejiang, 316000, China

^4^ ENN (Zhou Shan) Natural Gas Pipelines Co., Ltd, Zhoushan, Zhejiang, 316021, China.

* Corresponding Author at: Zhejiang Key Laboratory of Petrochemical Environmental Pollution Control, National-Local Joint Engineering Laboratory of Harbor Oil and Gas Storage and Transportation Technology, School of Petrochemical Engineering and Environment, Zhejiang Ocean University, Zhoushan, Zhejiang 316022, China (Liangjun Gao)

E-mail: [gmndragon@163.com](mailto:gmndragon@163.com) (Liangjun Gao)


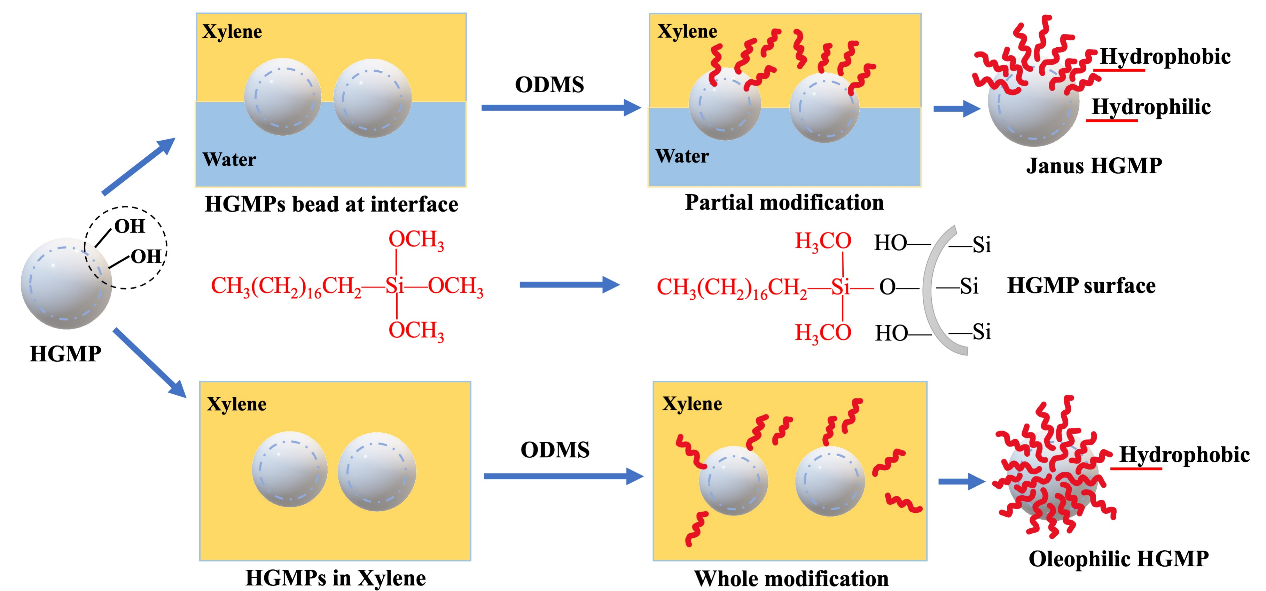


**Scheme S1.** Schematic illustration for synthesis of amphiphilic Janus HGMPs and oleophilic HGMPs.


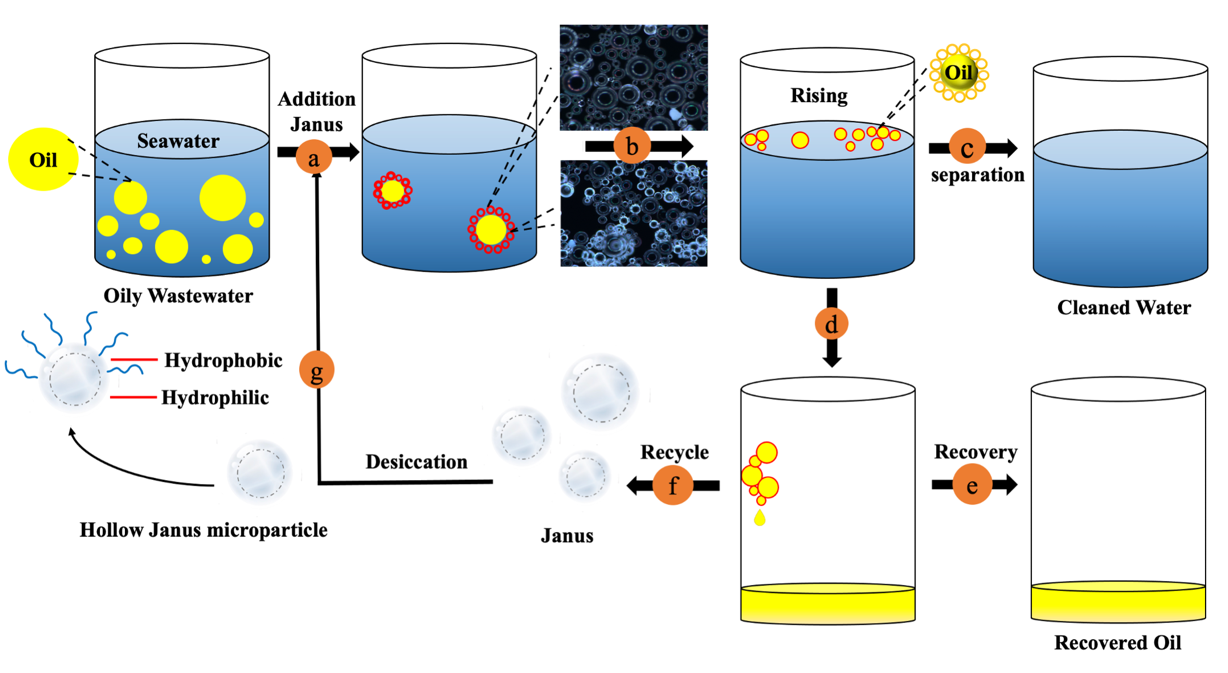
 **Schematic S2**. Schematic diagram of Janus HGMPs removing/recovering waste oil from oily wastewater.

When J-HGMPs are submerged in water and at the oil/water interface (Wu et al. 2022), respectively, the energy of a particle entirely in water, Ew (2), or at oil-water interface, E_interf_ (3):

$E_{w}= R^{2}(2{}_{w-pho}+2_{w-phi}+{}_{o-w})$ (2)

$E_{interf}= R^{2}(2{}_{O-pho}+2_{w-phi})$ (3)

For J-HGMPs, the *ΔG_1_* value of particles desorbing from the oil/water interface into oil phase can be obtained by Eq. (4):

Δ$G_{1}=E_{w}-E_{interf}= R^{2}(2{}_{w-pho}-2_{o-pho}+{}_{o-w})$ (4)

According to Young’s equation, γ_w-pho_ - γ_o-pho_ = γ_w_ Cosθ, Eq. (4) can be further expressed as:

Δ$G_{1}= R^{2}(2{}_{w}Cos{}_{1}+{}_{o-w})$ (5)

Similarly, for J-HGMPs, the ΔG2 value of particles desorbing from the oil/water interface into water phase can be obtained by Eq. (6):

Δ$G_{2}= R^{2}(2{}_{w}Cos{}_{2}+{}_{o-w})$ (6)

For above equations, *R* is the radius of J-HGMPs, *θ_2_* is the contact angle obtained via the water phase, and γ*_w_* , γ*_o-w_* , γ*_w-pho_* , γ*_o-pho_* , and γ*_w-phi_* are the surface tension of water, interfacial tension between of oil and water, water and the lipophilic part of J-HGMPs, oil and the lipophilic part of J-HGMPs, water and the hydrophilic face of J-HGMPs, respectively.
